# Incidence of abdominal pain due to the anterior cutaneous nerve entrapment syndrome in an emergency department

**DOI:** 10.1186/s13049-015-0096-0

**Published:** 2015-02-08

**Authors:** Tijmen van Assen, Jill A G M Brouns, Marc R Scheltinga, Rudi M Roumen

**Affiliations:** Department of Surgery, Máxima Medical Center, Eindhoven/Veldhoven, The Netherlands; SolviMáx, Center of Excellence for Abdominal Wall and Groin Pain, Máxima Medical Center, Eindhoven/Veldhoven, The Netherlands

**Keywords:** Intercostal neuralgia, Abdominal wall pain, ACNES, Emergency department, Incidence

## Abstract

**Background:**

Patients with chronic abdominal pain occasionally suffer from the anterior cutaneous nerve entrapment syndrome (ACNES). A substantial number of patients report previous visits to an emergency department (ED) with acute pain. Aim of this study was to obtain the incidence of ACNES in patients presenting with abdominal pain in the ED of a Dutch teaching hospital.

**Methods:**

In this observational study, data sets of all patients visiting Máxima Medical Center’s (MMC) ED in 2011–2012 were searched for key terms ‘ACNES’, ‘intercostal neuralgia’ or ‘abdominal wall pain’. Files of potential patients living within hospital’s adherence area were checked using accepted criteria indicating the presence of ACNES. Disease incidence was calculated as a ratio to the hospital’s adherence data. The ACNES MMC 2013’s incidence in patients evaluated in the surgical outpatient department was also calculated.

**Results:**

During the study period, 473 ED patient files met inclusion criteria. A total of 88 patients belonging to MMC’s adherence area were diagnosed with ACNES leading to a mean 22/100.000 ACNES yearly incidence rate. About one of 50 patients with abdominal pain visiting the ED suffered from ACNES. A 35/100.000 outpatient department ACNES incidence rate was calculated in the year of 2013. Combining these two ratios, a 1:1800 ACNES incidence in the general population was obtained.

**Conclusions:**

In an ED setting of a Dutch teaching hospital, approximately 2% of patients presenting with acute abdominal pain suffered from ACNES. ED physicians should consider ACNES in abdominal pain patients with normal laboratory or imaging tests.

## Background

Patients with abdominal pain may suffer from an abdominal *wall* related entity. A typical example is the anterior cutaneous nerve entrapment syndrome (ACNES) [1-9]. End branches of thoracic 8–12 intercostal nerves are caught in abdominal muscles causing severe neuropathic pain [2,3,6,10,11]. If recognized, injecting anaesthetic agents into the point of maximal pain is diagnostic and occasionally therapeutic [12,13]. If pain persists, removal of nerve end twigs may offer relief [12,14-17].

A literature survey on incidence of acute abdominal pain in a general emergency department (ED) demonstrated that approximately 1% of referrals to general surgeons were related to these acute abdominal wall pain syndromes [9]. Moreover, 20% of patients who presented to the ED with a nonspecific acute abdominal pain tested positive for abdominal wall tenderness suggesting the presence of an abdominal wall pain syndrome [18]. However, such estimations are biased by author selection and preference. Therefore, a reliable estimation of incidence of acute abdominal wall pain syndromes such as acute ACNES presenting to an ED is not available.

Physicians working in the SolviMáx centre for abdominal wall and groin pain syndromes are experienced in diagnosing ACNES in adults and adolescents [6,11-17,19]. During outpatient department intakes, these ACNES patients frequently reported on earlier ED visits with attacks of abdominal pain. Unfortunately, pain was then often not recognized as due to nerve entrapment leading to a substantial doctor’s delay [2,7,20]. With this finding in mind, awareness in an ED regarding ACNES may seem suboptimal. However, if a triad of abdominal pain, a circumscript pain point (in the presence of a positive Carnett test and/or local sensory disturbances) and a normal blood analysis or ultrasonography/computed tomography are present, a person may suffer from this typical ‘clinical syndrome’.

Aim of the present study was to calculate the incidence of ACNES in the ED of a Dutch teaching hospital. If substantial, ACNES must attain a more prominent position on the list of differential diagnoses of acute abdominal pain in an ED.

## Methods

### General information

Data of this retrospective observational study was obtained between 2011 and 2014 in Máxima Medical Centre (MMC), a 543-bed community hospital situated in the southern part of The Netherlands serving a population of approximately 200.000 inhabitants. MMC is considered a typical example of an average Dutch teaching hospital. Approximately 30 thousand patients present to its ED each year. Comparable to published numbers, some 8 percent of visits to MMC’s emergency department is due to abdominal pain [21-23]. Each abdominal pain patient is standardly evaluated by interns of the ED, surgery or internal medicine. If deemed necessary, patients are subsequently evaluated by a senior emergency physician or by a consultant (emergency medicine, surgery, internal medicine, gastroenterology, urology etc.).

A surgical subdepartment - separate from the ED - has gained ample experience in the evaluation of patients with chronic abdominal wall pain and groin pain syndromes (‘SolviMáx’ Center of excellence for Abdominal Wall and Groin Pain) [13,14,24]. MMC physicians are regularly updated on new evidence regarding ACNES by these SolviMáx specialists. Therefore, awareness on our ED regarding ACNES is likely higher than ED’s of other (Dutch) hospitals.

The MMC Medical Ethical Committee concluded that the rules laid down in Medical Research Involving Human Subjects Act did not apply to the present research proposal and hereby gave permission to perform the study (N15-004).

### Data collection

Electronically stored data using the ChipSoft© health and information system (CS-EHIS, version 5.2 HF188, ChipSoft B.V., The Netherlands) served as the source for the present study. This software program is used by all MMC health care providers for documentation of patient visits [25]. Two sets of data were extracted from this CS-EHIS system. The first set contained data of all patients who visited the MMC’s ED in the year of 2011 and 2012. The second dataset consisted of surgical patient records containing one of the following keywords: ‘ACNES’, ‘intercostal neuralgia’ or ‘abdominal wall pain’. By electronically combining these two data sets, patients of interest were identified and were inserted in a separate data file termed ‘potential ACNES-ED patient file (ACNES-EDPF)’ that was used as the basis for the present study.

As ACNES is generally considered a typical clinical syndrome, a diagnostic gold standard is currently lacking. However, the diagnosis was deemed likely in a patient if the following set of signs and symptoms, as registered in CS-EHIS, were met:

1. Presenting with abdominal pain at the ED, and
2. Site of abdominal tenderness with a small (<2 cm^2^, “finger tip”) area of maximal intensity (trigger point) situated within lateral boundaries of the rectus abdominis muscle, and
3. Normal laboratory analysis and normal imaging (if performed for this specific ED visit), and
4. Pain reduction following infiltration using 5–10 mL of 1% lidocaine, and
5. The diagnosis ACNES was confirmed during a follow up ED visit, or shortly thereafter during a visit to one of MMC’s surgical outpatient departments by one of the SolviMáx pain specialists as reflected in the surgical patient record.

**Figure 1:**
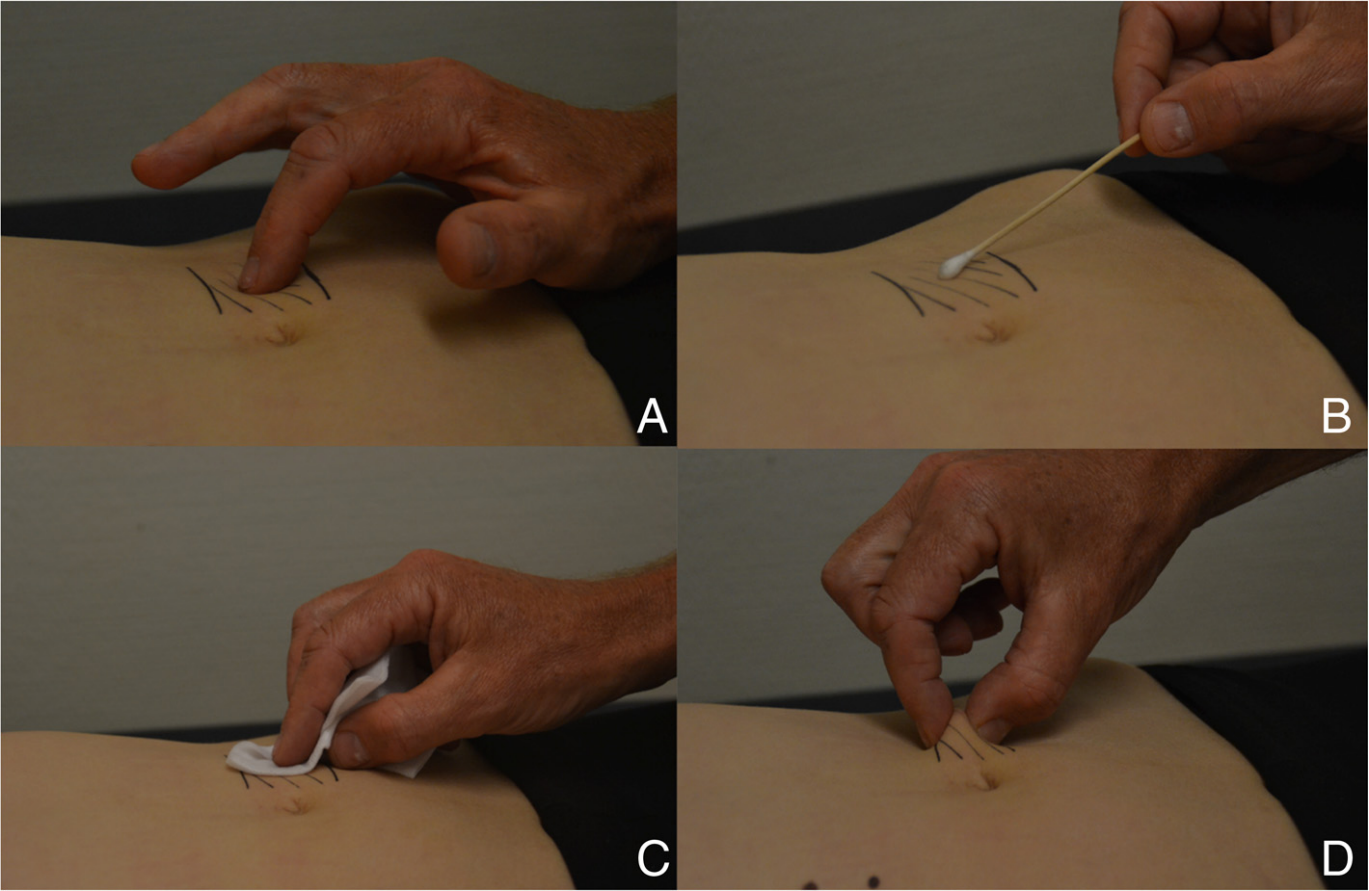
**Typical findings during physical examination in an ACNES patient. A**. Trigger point is localized using palpating index finger. Tenderness increases when abdominal muscles are tensed as the patient lifts the head (Carnett’s test); **B**. Skin gnostic sensibility is determined using a swab. The shaded area overlying the trigger point reflects altered sensibility; **C**. A cold alcohol gauze is utilized to test the vital sensibility; **D**. By squeezing a fold containing the patient’s skin and subcutaneous fat between thumb and index finger, the area of interest hurts compared to the contralateral side (‘pinch test’).

If the diagnosis in an individual that was present in this ACNES-EDPF file was deemed uncertain, history and physical examination, as well as diagnostic and therapeutic measures that were tabulated in the patients’ file were again checked for additional specifics and clues. If still in doubt, one of the SolviMáx pain specialists (n = 4) was consulted for advice. Typical findings such as a positive Carnett’s test, a positive pinch test and the presence of local sensory disturbances or lateral/paravertebral trigger points strongly contributed to confirmation of the diagnosis (Figure 1A-D) [2,3].

Known ACNES patients who visited the ED were excluded. Patients who were suspected of having ACNES whereas history and findings at physical examination were not consistent with the syndrome were also excluded. These patients were usually referred to the ED by family physicians. To correct for spectrum bias, individuals living outside MMC’s adherence area were excluded as well.

Accurate figures on MMC adherence were obtained from a market analysis. In the year of 2011, a total of 206.000 individuals were assumed to visit the MMC for medical assistance. In the year of 2012, this adherence number was officially set at 199.000 residents (Kiwa Carity B.V., Utrecht, The Netherlands). These numbers are considered to be an optimal approximation of MMC’s real adherence.

An additional calculation of the 2013 incidence of chronic ACNES was based on first time patient visits to the SolviMáx outpatient department. Each first SolviMáx outpatient department visit of an individual referred as possibly having ACNES is prospectively registered from January 1, 2013. Electronic patient files of all individuals diagnosed with ACNES via this system were also studied. Patients were excluded if they lived outside of MMC’s adherence area, if they were known ACNES patients, or if they had presented to MMC’s ED in an earlier stage (2013 MMC adherence, approximately 200.000).

### Data analysis

Data analysis was performed using SPSS 21.0 for Mac OS X (IBM SPSS statistics, IBM corporation, New York, United States). Baseline characteristics were presented as percentages, mean (SD), or median values (range) as appropriate. Adherence numbers of MMC and numbers of ACNES patients diagnosed in 2011 and 2012 were used to calculate an ACNES ED incidence and a mean incidence rate using standard calculus.

## Results

### Incidence of ACNES on ED in 2011 and 2012

In the year of 2011, 31.325 patients attended the ED of MMC (2012: 30.023). In the 2-year study period, 5.111 patient visits were registered for abdominal pain (8.3%, 5111/61.348). A total of 473 ED patient files harboured the preselected keywords ‘ACNES’, ‘intercostal neuralgia’ or ‘abdominal wall pain’. A total of 186 patients (39%, Table 1) was also identified via these keywords. However, they were excluded as they visited the ED for a non-abdominal illness (n = 186).

**Table 1:** **Patients potentially having ACNES visiting the emergency department of a Dutch teaching hospital in 2011-2012**

	**2011**	**2012**	**2011/2012**
Patient file containing key words ‘ACNES’, ‘intercostal neuralgia’ or ‘abdominal wall pain’ (n)	250	223	473
Presenting to ED with a non abdominal illness (n)	114	72	186
*Trauma*	*52*	*27*	*79*
*Fever/malaise*	*19*	*6*	*25*
*Pulmonary*	*10*	*5*	*15*
*Cardiac*	*9*	*11*	*20*
*Neurological*	*2*	*3*	*5*
*Skin infection and/or abscess*	*7*	*5*	*12*
*Collapse*	*1*	*3*	*4*
*Other*	*14*	*12*	*26*
Presenting with abdominal pain (n)	136	151	287
ACNES (n)	41	56	97
Excluding patients outside MMC adherence area (n)	−6	−3	−9
Diagnosis ACNES within MMC adherence area (n)	35	53	88

In 2011, 41 ED patients with (acute) abdominal pain were diagnosed with ACNES, the majority (n = 35) belonging to MMC adherence area (Table 1, left hand panel). As 206.000 registered individuals adhered to the MMC area, a 17/100.000 incidence rate was calculated. A similar calculation regarding the year of 2012 (56 ACNES, 53 in a 199.000 MMC adherence) yielded a 27/100.000 incidence rate. As a consequence, a mean 22/100.000 ACNES ED incidence rate per year was obtained over these two years. If all 5.111 patient visits with abdominal entities in the ED were considered, approximately 1:50 was found to have ACNES.

Baseline characteristics, history and findings of physical examination in this population of 88 ACNES patients are summarized in table 2. The majority was female (n = 71, 81%), and median age was 37 years (range 12–76). Most (n = 62, 70%) patients reported that the pain had started in the previous week. A typical right lower abdominal preponderance (59%, n = 52) was found.

**Table 2:** **Characteristics of patients diagnosed with ACNES (n = 88) in a Dutch emergency department during a two year period (2011/2012)**

**Age, median (range)**	**37 (12–76)**
Gender (M:F)	17:71
Duration of symptoms	
<1 week	*62*
1 week – 1 month	*14*
1 month – 6 months	*10*
>6 months	*2*
**Physical examination**	
Abdominal pain location, n	
Right upper quadrant	*13*
Left upper	*5*
Right lower	*52*
Left lower	*17*
Bilateral lower	*1*
Trigger point, Yes: No (missing data, n)	88:0 (0)
Carnett-sign, Pos: Neg (missing)	65:5 (18)
Sensory disturbances, Y: N (missing)	60:9 (19)
Pinch, Pos: Neg (missing)	37:5 (46)

### 2013 outpatient department ACNES incidence

In the year of 2013, a total of 268 new onset patients visiting the SolviMáx outpatient department were diagnosed with ACNES. However, just a small portion (85/268, 32%) belonged to the adherence area of MMC confirming its status as a referral centre for the remaining 68%. As 15 of these 85 newly diagnosed ACNES patients had attended the ED of MMC in an earlier phase, a 35/100.000 incidence rate of ACNES in the outpatient department was calculated. By combining numbers of ED and outpatient department, a total 57/100.000 ACNES incidence rate was obtained.

## Discussion

It is our experience that some patients who are ultimately diagnosed with chronic abdominal pain due to the anterior cutaneous nerve entrapment syndrome (ACNES) reported that they had previously attended emergency departments (ED) for earlier attacks of pain. As blood analysis, ultrasounds or CT-scans were always inconclusive, they were frequently sent home without receiving a conclusive diagnosis resulting in a substantial doctor’s delay. To this day, ACNES is consistently neglected as a cause of abdominal pain [2,5,7,8]. Awareness regarding ACNES as part of the differential diagnosis of *chronic* abdominal pain in our area is high [6,11-17,19]. It may be assumed that the chances of recognizing ACNES in patients with *acute* abdominal pain visiting our ED are also higher. Aim of the present study was to calculate the incidence of ACNES in the ED of a Dutch teaching hospital. Based on an analysis of all patients visiting during the 2011/2012 time period, a mean 22/100.000 per year incidence rate was obtained. In perspective, about one of 50 patients presenting with abdominal pain to the ED was diagnosed to suffer from ACNES. In an ED such as MMC’s hosting approximately 30 thousand patients yearly, the diagnosis ACNES is made almost once a week. An emergency physician must realize that a patient who is referred to the ED for abdominal pain of unknown origin may suffer from an abdominal wall pain syndrome such as ACNES.

Most patients with chronic abdominal pain due to ACNES are diagnosed after a substantial time period. For instance, mean doctor’s delay was more than a year in one study indicating that the pain is per definition chronic [13]. Interestingly, most patients in the current study (70%) presented to the ED within the first week following the onset of pain. Although generally considered a chronic entity, a substantial portion of ACNES patients apparently experiences pain in an acute setting. This novel finding clearly illustrates our limited knowledge on various issues regarding the syndrome. As a consequence, most patients with an acute ACNES syndrome probably move on to develop a chronic form due to insufficient awareness of consulted doctors.

How can consciousness regarding signs characterizing ACNES be increased? A physical examination has a pivotal role in the diagnosis. If serious causes of acute abdominal pain are excluded in the ED using a combination of physical examination, blood analysis and imaging techniques, results of simple tests may point towards the presence of ACNES. A positive Carnett’s test and an area of sensory disturbances covering the painful spot are signs reflecting the neuropathic character of this pain syndrome [2,7,10,18,26,27]. We routinely evaluate skin gnostic sensibility using a simple swab. Hypoesthesia, hyperesthesia/algesia or sometimes even allodynia may be found by comparison to the normal contralateral abdominal side. In addition, a gauze soaked with alcohol is utilized to test vital (cold) sensibility. A positive pinch test is a sensitive and highly underrated sign reflecting the presence of a neuropathic pain syndrome [2]. By squeezing a patient’s skin fold and subcutaneous fat using thumb and index finger, the area of interest will hurt when compared to the contralateral side (Figure 1). The majority of patients (88%, 37/42) in the present study tested positive. A skin pinch test deserves a standard place in the ED physician’s toolbox of physical examinational tests for abdominal pain.

When the diagnosis ACNES is likely, ED physicians are advised to start trigger point injection therapy. A subfascial injection of 5–10 ml of 1–2% lidocaine leading to immediate pain reduction is diagnostic and occasionally therapeutic. Previous studies in chronic ACNES demonstrated permanent pain relief in one-thirds of the patients after injection therapy [12,13]. Patients with recalcitrant pain after repetitive injections are offered resection of entrapped branches of the intercostal nerve leading to long-term pain remission in up to three-quarters [12,16]. In the remaining population, a renewed surgical exploration is found to be effective in an additional two-thirds of the patients. This three-step treatment protocol may lead to pain remission in some ninety percent [17]. Interestingly, the response to immediate injection therapy may be more favourable in acute ACNES patients. For instance, a conservative strategy sufficed in the majority (64/88, 73%) of the present study population. Conversely, just 24 patients (27%) in the study population underwent a neurectomy comparing favourably to a 50% surgical rate as reported in another group of chronic patients who were still painful after injection therapy [12]. An optimized response to injection therapy in acute ACNES may be related to the short duration of complaints. This hypothesis is currently tested in our patient population with ACNES.

How does the present ACNES incidence relate to other known disease entities in the Netherlands? The calculated 57/100.000 (1:1800) total ACNES incidence is obtained in our area containing approximately 200.000 individuals. If extrapolated to the Dutch population (16.800.000 inhabitants), some 9.500 new ACNES patients may be identified yearly [28]. In a survey among alleged irritable bowel syndrome patients in a general practice setting in the Netherlands, a prevalence of some 20 thousand ACNES patients (being approximately 4% in relative numbers) within this functional abdominal pain population was calculated [11]. The 9.500 ACNES incidence yearly exceeds numbers of Dutch patients that are newly diagnosed with for instance inflammatory bowel disease (n = 7.200) or stomach and duodenum ulcer (n = 6.700) [29]. The incidence number of ACNES patients visiting the ED is calculated at 1:4.500 (22:100.000). It is interesting to relate these numbers to for instance the well-known incidence of acute appendicitis (approximately 1:1.000). For every 4–5 acute appendicitis patients, one additional individual with acute abdominal pain is diagnosed with ACNES [30]. From a different perspective, as an average Dutch general practice contains about 2500 patients, a Dutch general practitioner will be confronted with 1–2 new ACNES patients yearly.

The current study harbours flaws associated with its observational character including incomplete data sets. For instance, some specific characteristics of history taking and physical examination were not routinely registered possibly indicating bias. Moreover, the electronic searching strategy may have been incomplete as the number of used key words in the CS-EHIS may have been insufficient or incorrectly registered by ED physicians. Although these effects are considered minimal, an incidence underestimation rather than an overestimation may have resulted from these flaws. Another disadvantage is potentially related to the institution where this study is executed. Although groups of physicians working on MMC’s ED are rotating, there is no doubt that awareness regarding this neglected syndrome is high compared to other ED’s. Therefore, there may be a risk of ‘overdiagnosing’ patients with abdominal wall pain syndromes, also because the diagnosis ACNES is a typical clinical diagnosis lacking a gold standard. However, we strive to strictly adhere to objective criteria. Moreover, no alternative abdominal diagnoses emerged following presentation at the ED or during follow up.

## Conclusion

Approximately 2% of all patients presenting with acute abdominal pain were diagnosed with anterior cutaneous nerve entrapment syndrome (ACNES) in an emergency department setting. If patients with a localized acute abdominal syndrome demonstrate normal laboratory or imaging testing, ED physicians should consider ACNES in the differential diagnosis.

## Abbreviations

ACNES: Anterior cutaneous nerve entrapment syndrome
ED: Emergency department
MMC: Máxima medical center
CS-EHIS: ChipSoft© health and information system
ACNES-EDPF: Anterior cutaneous nerve entrapment syndrome – emergency department patient file
SD: Standard deviation
